# Multimodal artistic metaphors: Research on a corpus of Sardinian art

**DOI:** 10.3389/fpsyg.2023.1146639

**Published:** 2023-09-07

**Authors:** Alice Guerrieri, Francesca Ervas, Elisabetta Gola

**Affiliations:** ^1^Department of Education, Psychology, Philosophy, University of Cagliari, Cagliari, Italy; ^2^Faculty of Communication, Culture and Society, University of Italian Switzerland, Lugano, Switzerland

**Keywords:** artistic metaphors, figurative language, multimodality, metaphor, Sardinia

## Abstract

The study aims to define an artistic metaphor and highlight the multimodal properties of metaphors in artistic environments. In this research, an artistic metaphor has a relevant aesthetic purpose and it conveys beauty. Interpreting a metaphor in Sardinian art requires time for contemplation, however the cognitive effort of understanding the meaning of an artistic metaphor is rewarded by the delight of those who contemplate it. This metaphor has some characteristics in common with a visual metaphor but differs from other types of images that have been more extensively analyzed in the literature: it is difficult to establish a specific directionality, and consequently, it is not easy to recognize the target and source domains; the way it is expressed makes its interpretation and classification problematic at times. A proposal is presented in the paper to describe artistic metaphors according to universal macro-categories, inspired by the knowledge of Aristotelian and Kantian categories and by studies in the field of aesthetics: (1) time, (2) space, (3) decontextualization of stereotypes, and (4) fusion of forms. These categories are applied to a corpus of artworks by important artists in Sardinia to show that the visual, tactile, and auditory components of the pictures can boost an effective comprehension of figurative meaning. Results suggest that the multimodality of Sardinian artistic metaphors orients the observer toward original possibilities of learning and stimulates knowledge of the “submerged” wealth of symbols and archetypes that characterize insularity.

## 1. Introduction

Imaginative effects have been found in particular in the processing of metaphorical utterances and figurative ways of thinking. In *Philosophical Investigations* (1953), Wittgenstein contemplated the possibility of seeing and interpreting an image now as one thing and now as another (*sehen als*). For instance, the figure of Jastrow^1^ can be seen as either a hare's head or a duck's head (Wittgenstein, 1953). “Seeing as” is an intermediate concept between seeing and thinking; it is not to be understood as a state of an exclusively intellectual nature (Voltolini, 1998; p. 144) and differs from the ordinary perception of an object because it has a voluntary aspect.

According to Davidson (1978), metaphorical language does not evoke concepts that are linguistically encoded but rather images that are not part of the encoded language. As a secondary effect of linguistic and pragmatic processing, the recipient often imagines visual content in their mind that is automatically activated *via* mental imagery.

Mental images are non-propositional effects of language (Carston, 2018). More specifically, this type of figurative or literary language is characterized by a variety of expressive nuances, their undefined aspect, and the possibility of their triggering perceptual, emotional, and sensorimotor processes (Wilson and Carston, 2019). Although they retain a trace of the literal meaning, imaginative effects are especially formed in the process of interpreting novel verbal metaphors (Carston, 2010). In addition, some properties that have been ignored or undervalued in the involved domains of metaphor may be highlighted when mental images are evoked (Indurkhya, 2006, 2016; Wilson and Carston, 2008), and also in the visual sphere (Bolognesi and Cavazzana, 2020).

When comprehending visual metaphors, multimodal mental imagery (Nanay, 2016) acts similarly to an inference in the verbal realm; it may be a “seeing” or a “hearing” despite the lack of appropriate immediate sensory input and is distinct from perception, which is instead the registration of a physically present stimulus (Kosslyn et al., 1995). Unlike compulsory and involuntary perceptual processing, the production of mental imagery derives from memory and the ability to imagine and acts under the control of the will (in the sense that it can be activated or not). Visual metaphors in advertising communication, which involve quick comprehension, often activate the observer's mental image.

**Figure 1:**
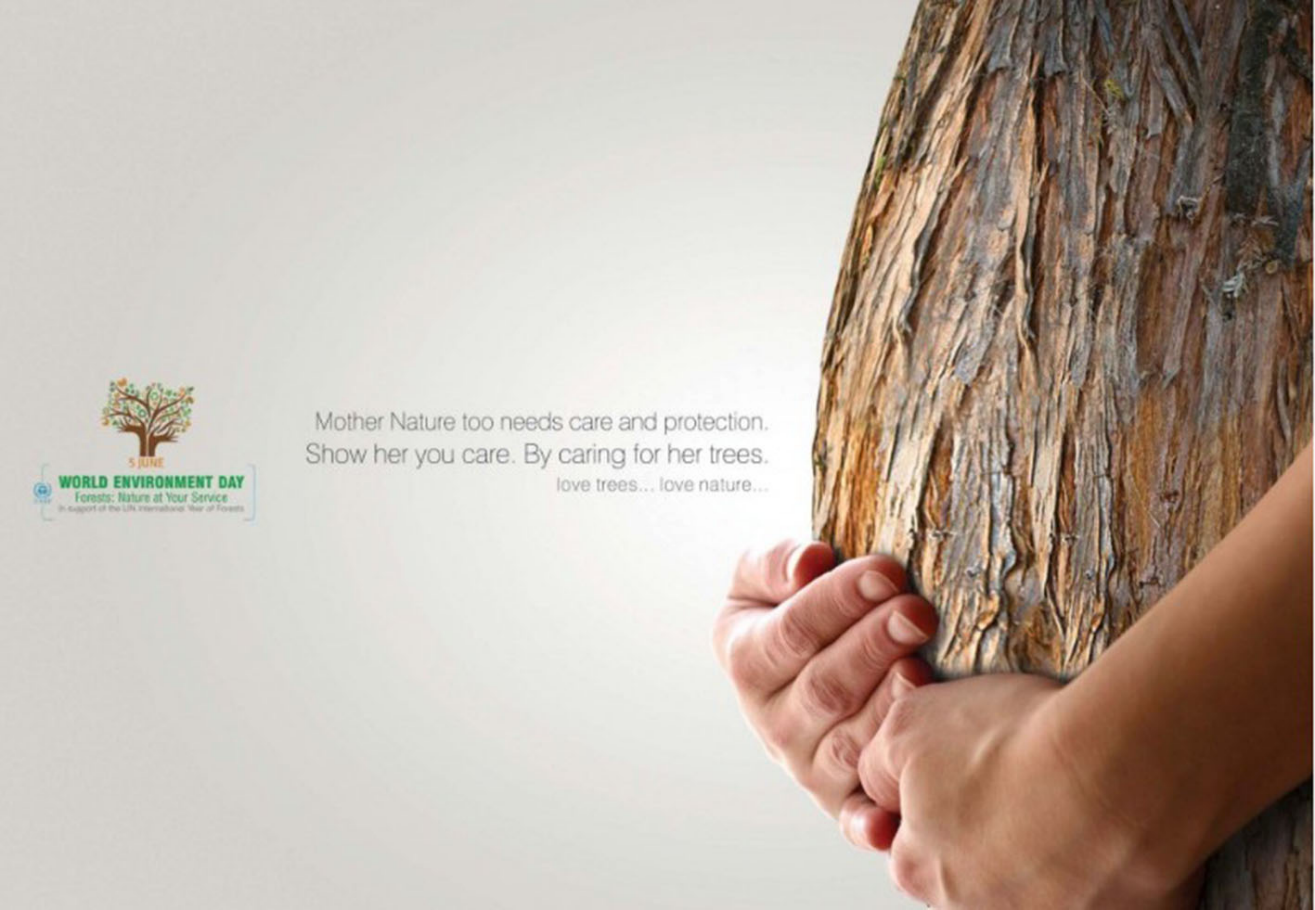
Example of contextual visual metaphor (World Environment Day, reproduced with permission from Creative agency: Valappila Communication, India).

The triggering of mental imagery is necessary to understand those visual metaphors in which one of the two domains is not visible. For instance, in the contextual visual metaphor, the image-demanding metaphor in Figure 1 requires an image of not only the object representing the source domain (the pregnant belly), but also the metaphorical action of caressing the pregnant belly (instead of embracing the tree) (Ervas, 2019).

Some visual metaphors are also used in education due to their communicative effectiveness. These metaphors show a vividness that carries new meanings and has a profound impact on our experience of the world (Contini and Giuliani, 2021; p. 10).

It is now necessary to define what is meant by artistic metaphors. In this research, an artistic metaphor has a relevant aesthetic purpose: it conveys beauty and enjoyment. Interpreting an artistic metaphor, in this particular case a metaphor in Sardinian art, requires time for contemplation and activation of the senses. However, the cognitive effort of interpreting the meaning of an artistic metaphor is rewarded by the delight of those who contemplate it. What is perceived is not neutral: it can imprint itself on the observer's memory, reinforce cultural identity, and trigger universally applicable thoughts.

An artistic metaphor has some characteristics in common with a visual metaphor: it can be a *monomodal metaphor* when the target and source domains are equally represented in the same modality, as is often the case in untitled works of art in which the visual component is dominant; or it can be a *multimodal metaphor* when the target and the source are exclusively or predominantly represented in different modalities, which may include not only the visual and verbal modalities—as in the title of the work or in verbal utterances placed within the visual framework, such as in street art—but also the acoustic, tactile, and olfactory modalities (Forceville and Urios-Aparisi, 2009).

An artistic metaphor can meet the conditions of homospatiality and non-compossibility (Carroll, 1994). It can show incongruent effects in the image and trigger surprising thought processes in the viewer. However, an artistic metaphor differs from other types of images that have been more extensively analyzed in the literature for the following reasons: (1) It is difficult to establish a specific directionality, and consequently, it is not easy to establish the target and source domains; (2) The way it is expressed through the realization techniques and color rendering makes its interpretation and classification problematic at times; and (3) The artist who creates an artistic metaphor often does not have to pursue a commercial goal (like an advertising graphic designer) and, therefore, projects their way of imagining and interpreting reality onto the image they create, possibly disorienting the interpreter in the stages of decoding meanings.

Finally, an artistic metaphor is different from metaphorical art: in an artistic metaphor, the metaphor is found in the visual structure of the artwork, whereas in metaphorical art, the metaphor can only be found in the titling of the artwork. For instance, in *The River of Life*, a work of landscape painting by William Blake,^2^ the metaphor is only found in the caption (the textual content is conceptualized by the metaphor *Life is a river*) and is not found in the visual structure of the artwork (Wollheim, 1984).

In what follows, nine works of art produced on the island of Sardinia (Italy) are analyzed to show that artistic multimodality can orient the gaze of the recipient toward new perspectives of understanding.

## 2. Categorization of metaphors in art

Forceville (1996, 2008) categorized visual metaphors into four types:

1. *Hybrid visual metaphor*: when the objects representing the source and target domains are fused into a single object.
2. *Contextual visual metaphor*: when the object representing the source substitutes for the object representing the target or vice versa.
3. *Pictorial simile*: when the source and target domains are depicted by juxtaposed objects.
4. *Integrated metaphor*: when the object is represented in its entirety so that it resembles another object even without contextual cues.

Bolognesi et al. (2018) also cataloged a genre-structured corpus, taking into account the denotative and connotative dimensions of meaning. Observing the corpus, it seems more effective to frame images in specific categories that belong to advertising communication in specific categories. Indeed, target domains are easily distinguished from source domains because they reflect the promotional intentions of brands, and visual structures are persuasively devised because they are intended to achieve specific commercial purposes. Therefore, all “other” images that cannot be placed within a specific visual scheme need a different framing. Artistic metaphors are not often bound to such specific objectives to be pursued by the artist. Rather, these metaphors manifest a certain freedom of expression in the use of techniques, color rendering, and representation of subjects, suggesting multiple levels of interpretation to the interpreter. Therefore, a new and systematic categorization of artistic metaphors is needed.

## 3. Methodology

A proposal is presented in the study to describe artistic metaphors according to universal macro-categories, inspired by the knowledge of Aristotelian and Kantian categories and by studies in the field of aesthetics and philosophy of art (Walton, 1970): (1) time, (2) space, (3) decontextualization of stereotypes, and (4) fusion of forms (Table 1).

**Table 1:** Four categories to describe artistic metaphors.

Time	• In the creation (e.g., *Balkan Baroque*, Marina Abramović) • Immediacy (e.g., *The Artist Is Present*, Marina Abramović) • Point of view of the observer (e.g., *Stories of St. Francis*, Giotto) • Inside/outside the frame • With/without a caption
Space	• Inside/outside the frame (e.g., murals, Keith Haring) • Space within space (e.g., *La condition humaine I, II*, René Magritte) • Symbolic (e.g., *The Scream*, Edvard Munch) • With/without a caption
Decontextualization of stereotypes	• Surreal dimension (e.g., *The Persistence of Memory*, Salvador Dalí) • Inside the frame (e.g., *Guernica*, Pablo Picasso) • Outside the frame with unusual techniques of realization (e.g., *Sacco Rosso*, Alberto Burri) • With/without a caption
Fusion of forms	• Rendered using expressive techniques (e.g., *The Starry Night*, Vincent van Gogh) • Expressed through physicality (e.g., *The Broken Column*, Frida Kahlo) • With/without a caption

Only four categories are discussed: the category of decontextualization of stereotypes and one of the fusions of forms that, though universally recognized (such as those of space and time), have cultural implications. These categories help us identify the multimodal structure of artworks. The first macro-category is “time,” which is divided into various sub-categories. Temporality is inherent in the creation of the artwork. For instance, performance works that need a time frame to be realized and/or to manifest themselves unexpectedly to the spectator. In the performance *Balkan Baroque*, Marina Abramović sat for 6 hours a day for 4 days on a pile of cattle bones, which she cleaned of flesh and cartilage to denounce the atrocities of the Balkan war.^3^ The physicality shown and the metaphorical representation of the bodies (Wollheim, 1984) allow the observer to fully understand the meaning of the provocative action: the bones torn from the flesh of cattle can evoke in the mind of the beholder the atrocious imagery of people who died in the war (Settis and Montanari, 2019; p. 400). Works that arise only in a temporal dimension of immediacy are also available. The performance *The Artist Is Present*^4^ in 2010 was generated when Abramović sat at a table and looked at the visitors to the MoMA in New York, activating unexpected perceptual experiences in herself and the audience. Thus, the temporal dimension can be expressed not only from the viewpoint of the artist who creates the work but also from that of the viewer. In other words, some artistic objects require a specific time of enjoyment to understand their innermost meaning. This is the case with the cycle of frescoes dedicated to the *Stories of St. Francis* painted by Giotto at the end of the 13th century in the Upper Basilica of Assisi, which is structured as a sacred representation with edifying effects that are articulated during the time of contemplative observation by the faithful.

In the fresco illustrating the *Dream of St. Francis*,^5^ the saint (on the left of the image) is sleeping in a four-poster bed; next to him is an angel suggesting that he directs his gaze to the right, where an enormous palace with precious military friezes has appeared. The vision of the saint could be understood metaphorically and correspond to the celestial kingdom. The interpretation of this episode requires meditative observation time and knowledge of the events of the saint's life. The image also shows a temporal relationship that develops between an earlier situation (when the saint falls asleep) and a later one (the dream of the palace) (Girardi, 1999; pp. 38–39).

The abstract concept of time is expressed in metaphorical terms connected to the idea of space. Indeed, the metaphors of space are used to express the sense of time not only in the verbal but also in the visual and multimodal realms. In the decontextualized landscape of the work *The Persistence of Memory* painted by Salvador Dalí,^6^ three clocks appear in a state of liquefaction, marking a different time that does not flow in a coherent manner. The “hyper-liquidity” of the clocks (inspired by the observation of melted Camembert cheese) symbolizes the four-dimensional space–time continuum envisaged by the theory of relativity. In such a theory, each body possesses its own time based on its movement and energy state and not on that measured by clocks, thus conforming to the energy states occasionally assumed by the body itself. If man-made time is transitory, then the “persistence of memory” that lies in the illuminated background of the rocky shores can survive (Weyers, 1999; pp. 28–29; Nicosia, 2002; p. 43).

The frame delimits the spaces of observation, inside and outside, and “separates the image from all that is non-image. It defines what it frames as the signifying world, as opposed to the outside frame, which is the world of simple experience” (Stoichita, 2018; p. 173). The observer can venture with their imagination beyond the limits of the frame until they cross the boundaries of the gaze and the threshold of an entirely new dimension. In fact, the second macro-category of “space” and its sub-categories focus on existing artworks within the limits of the frame and those that go beyond them to include the places of creation themselves. Examples of the latter are murals, often created to redevelop urban environments and public spaces, as in the art of Keith Haring,^7^ or spaces redesigned through the participation of the audience, which is both the subject and object of the work of art along with the artist. The theme of the picture within the picture defines the space within the space, marking the boundary between reality and representation and between thinking in images and seeing. The painted image is an appearance that deceives the eye by passing itself off as the reality it represents, and the representation is substantially different from what is represented. Many examples come from the works of René Magritte,^8^ which symbolically illustrate the condition of a person living in a misleading world as if suspended between real and illusory space (Argan, 1990; p. 379. Paquet, 2001; p. 67). Space is decontextualized and distorted in its features, evoking dreamlike images (as in *The Persistence of Memory*) and becoming a metaphor for human anxieties [as in Edvard Munch's *The Scream*.^9^ (Settis and Montanari, 2019; p. 44)]

The third macro-category illustrates the subversion of stereotypical thinking when it is removed from its usual context. The use of stereotypes, that is, a set of certain properties characterizing a class of individuals, objects, or actions, is a way of categorizing reality and can help understand complicated situations (Domaneschi and Penco, 2016; p. 5). Therefore, “decontextualization of stereotypes” can subvert a pre-constituted way of thinking when the depiction shows a different place from the ordinary one known in our minds. Since the attribution of the meaning of a term implies the cooperation of a linguistic community and knowledge of the real world (Putnam, 1975), stereotypes, which are also conveyed through multimodality, can be misunderstood and lead us to errors in evaluation or reasoning, especially when they create a barrier to specific social categories. For instance, *The Persistence of Memory* subverts the stereotypical idea of time as a quantifiable and measurable category according to ordinary human rhythms. It intensifies unreal aspects in a surreal context. The subversion of stereotypes can be observed within the limits of the frame (understood as a sub-category), as in Pablo Picasso's *Guernica*,^10^ which sets the horrors of the bombing of the city of Guernica (Argan and Oliva, 2017; pp. 233–236) and the lacerations of bodies in a domestic interior rather than on the war front, as expected based on our knowledge of the world. Decontextualization can also be expressed with unusual techniques of realization; for instance, re-used materials that mark the overcoming of the limits imposed by frames (understood as a sub-category), in which substance becomes space and space its opposition (Argan, 1990; p. 483, Argan and Oliva, 2017; p. 297), as in those works by Alberto Burri made by juxtaposing acrylic colors and jute.^11^

The fourth macro-category is the “fusion of forms”, which is achieved through means of expressive technique or the use of color and can be compared to Forceville's fusion category (hybrid metaphor), in which the elements of the images are merged (see also Phillips and McQuarrie, 2004). In Vincent van Gogh's *The Starry Night*,^12^ the metaphorical representation of the stars rotating similar to endless whirlpools is achieved by the vibrant brushstrokes through a pictorial material that radiates in a circular manner and almost contrastingly blends ultramarine, cobalt blues (the hues of the sky), and Indian and zinc yellows (which tint the stars) (Cricco and Di Teodoro, 2012; pp. 1666–1669). The “fusion of forms” category is also expressed in corporeity, that is, the way of representing an image as an expression of emotions and thoughts about the body (Wollheim, 1984). In the work *The Broken Column*,^13^ Frida Kahlo depicts an ionic column ruined in several places to symbolize her diseased spine due to a serious accident. The gashes on her body, the chasms in the barren, and the lonely landscape merge to confirm the physical suffering of the artist (Kettenmann, 2001; pp. 68–69).

In the following section, the above categories and sub-categories are used on a corpus of works to effectively understand a specific socio-artistic context, such as that of the island of Sardinia, and show that multimodality is a useful tool for learning artistic techniques in addition to stimulating the imagination and inventiveness of the viewer.

## 4. Categorization of artistic metaphors: A case study

This case study focuses on the context of Sardinia, an island in the Mediterranean Sea that presents unique characteristics: the layered beauty of its landscape, the richness of its cultural identity heritage, and the caring generosity of its inhabitants. In this land, artistic language is characterized by its expressive originality, and the variety of techniques used by the artists enriches the viewer's cognitive experience. The artworks, indeed, were selected according to the following criteria: originality and evocative expressive potential in the variety of techniques used (sculpture, weaving, mural, and poly-material art, architecture, and illustration), presence of metaphorical domains, ability to stimulate the formation of mental images in the viewer, and ability to express meaning in the territory in which they are placed.

From our point of view, the categorization based on the four categories of time, space, decontextualization of stereotypes, and fusion of forms can increase the understanding of the most hidden meanings of the Sardinian artistic inheritance.

### 4.1. Time category

The sound sculptures of Pinuccio Sciola (1942–2016) reveal several features that lead to their classification in the category of “time” and its sub-categories. Stone is usually connotated negatively because of its properties of stiffness and clarity. However, Sciola enhanced its nature by transforming it into the temporality of work and converting it into a musical instrument. Just as the art of Socratic maieutics “extracts” from the souls and minds of the interlocutors those truths that they themselves were unaware of, so does Sciola with his stones. Silvano Tagliagambe wrote as follows:

“The artist does not impose on the stone a shape that he decides autonomously on the basis of his own intentions and choices but acts with cuts and incisions aimed at putting the material on which he is working in a condition to express its intrinsic potential to the fullest and in the most effective way, freeing the interior energy that it contains” (Tagliagambe, 2022; p. 142, our translation).

In the view of Sciola, the stone is to be understood as a human being and, therefore, can possess a soul and emit sparks when worked with the tool of the emery. When a stone is subjected to the heat of a blowtorch, its veins become so red-hot that they look similar to spurting blood. Such processes evoke the original conformations of basalts and expose primordial situations because weapons, cult stones, and shelters were built with stone.^14^

Therefore, the stones “contain” what the universe has produced since its very beginning. These stones are evidence of the geological vicissitudes of the island and become a metonymic tale of the earth itself because they are the guardians of ancestral sounds that have been articulated throughout history. The sounds can be “liquid” when emitted by limestone rocks or “deep” when coming from stones of volcanic origin.

**Figure 2:**
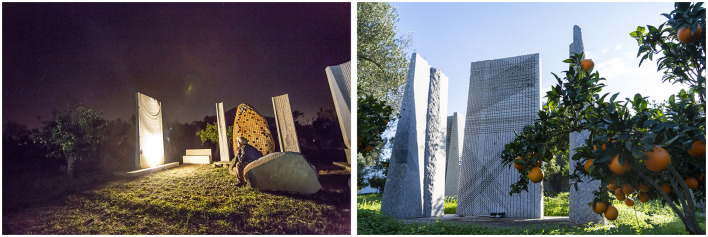
*Pinuccio Sciola* and his sculptures (reproduced with permission from Archivio Fondazione Sciola, ph. Ettore Cavalli).

Sciola's sculptures (Figure 2) are completely made in the place for which they are created and in the moment in which the artist plays them. Thus, a temporal *continuum* is established between what has been produced by history, the present of fruition, and the imagined future. Contemporaneity coexists “with its impetus toward the musical future; the prehistory with the archetypal and anthropological modes of meaning and communication through sound; the pre-cultural time of sonorous nature” (Favaro, 2011; p. 28, our translation) in these sculptures.

Non-verbal sound and music can play an important role in how a multimodal metaphor is structured and interpreted: these elements are useful in identifying the source domain of a metaphor, or they can serve to identify those features that are mapped from the source to the target (Forceville, 2009).

Considering an artistic metaphor in a sound sculpture, two domains are juxtaposed in an unusual manner: the stone could be understood as the source domain that the viewer can see, while the sound could be understood as the target domain, which is absent because it must be activated by the artist in this presence. In this way, the metaphorical component is so relevant that it enriches the interpretive process of the artistic work.

In fact, Sciola intentionally produces sonorities that stimulate the viewer's ways of observation and creativity (Wollheim, 1968). The comprehension of such a work occurs simultaneously with its instantaneous performance. The activation of the auditory imagistic component is necessary to reconstruct the meaning of the sculpture understood as an image-demanding metaphor, that is, a metaphor whose comprehension requires the activation of an image, which may be visual, tactile, or auditory (Green, 2017). As in the case of visual contextual metaphor (Forceville, 1996, 2002), string instruments such as a cello or harp are not displayed but instead suggested by the context in which the work is placed. The musical score generated by Sciola is also conveyed by the tactile component and the intimate experience the artist has with the stones^15^:

“The contact between the surface of the stone and the tactile surface of the fingertips and hands in all their extension, with their skin and its porosity between the outer and inner parts of our body, established a direct channel of communication that did not stop at the outer shell of the body itself, but, through the skin, penetrated inside, into the tissues and organs, extending to them the tactility of primary touch and involving them in this interaction” (Tagliagambe, 2022; pp. 118–119, our translation).

The sound sculptures are synaesthetic works that lead the performer to a multisensory experience capable of provoking a spiral of sensations, defined by the term *Gestaltkreis* (Read, 1956), from tactility to visual contemplation to auditory fruition (Ladogana, 2015; p. 11).

The time dimension of creating the work of art and observing it is also found in other works. In the production of the *Stitched Books* (*Libri Cuciti*) by Maria Lai (1919–2013), linguistic elements came from different fields: poetry, narration, and art. Time dedicated to the creation of the artwork and its observation can be found within the books. The artist redefines language through manual labor that is structured in the temporal dimension using a sewing machine. Maria Lai writes with a needle (instead of a pen) and thread (instead of ink); therefore, the fabric becomes a page, and the thread that joins the canvases becomes writing. The weaving is a visual score, that is, a rhythmic alphabet to be decoded with the eyes and the mind. The sewn books often drew their inspiration from a remote experience and the stories of an archaic civilization that had to be told and protected (Ciusa, 2017). The multimodality of these works is provided by the juxtaposition of “languages”; weaving meets writing, and the tactile component merges with the visual, making art performative (Alicata, 2019; p. 135). These works are imaginative experiences that have a considerable impact on the audience.

For instance, in the book *The Red Ants No. 5*,^16^ the threads are placed freely on the page, almost as if to suggest a way of understanding the vision of the textile movement as something different (the Wittgensteinian “seeing as”) (Wittgenstein, 1953). The observer does not find the known characteristics of a book and attempts to bridge the cognitive gap by activating an alternative image in the mind (visual mental imagery) (Nanay, 2016; p. 87) that enriches the creative experience of seeing. Thus, the usually printed character structure that identifies a book and that the observer expects to see represented is missing. The image of the book must be triggered by the occlusion shape (Hyman, 2006; pp. 75–76), which is generated by the tangled threads in the borders of the pages beyond the boundaries of the work (the source domain) and the typefaces of the book (the invisible target domain).

The tactile texture is occasionally a complex signifier to interpret, and a slow pace is required to read each page to decipher its deep meanings. Turning a cloth page is a slower gesture than turning a paper page or running the fingers over a screen. The consistency of the page can be felt between the fingertips (Anedda, 2019; p. 87).

Moreover, slowness evokes visions from the artist's past: as a child, she watched her grandmother mend the sheets. These threads that mended the tears fed her imagination and became narratives to be invented in the time frame of her story and ways of recreating childhood games.

*Holding the shadow by the hand*^17^ is a fairy tale made by sewing a thread onto pieces of cloth tied together to simulate the shape of a book. On the first page of the book, the caption “*holding the shadow by the hand*” is not only intended as a literal statement but also as a metaphorical expression, for its imaginative potential and the icastic power it evokes in the reader. This artwork narrates the formative journey of a “man-child”, as the author calls him, and illustrates a small pink rectangle with stylized eyes, legs, and arms. The protagonist of the story unfolds his life journey from page to page, stimulating the imagination of the reader with the characters he meets (himself as a playmate, shadowy enemies, and the blue demon angel) and surprising himself with his visions (the spaces of the fairy tale that take on color and the shadows that expand and then flow into a garden of flowers).

The work is understood as “the path of an awareness of the human being, the metaphor in a “situation” of looking at oneself in the mirror, of putting oneself into play, and opting for an act of freedom and courage” (Cuccu, 2014, our translation).

Two dimensions are present in the fairy tale: the temporal dimension of the flowing narrative and the dimension of reflective enjoyment on the part of the viewers, who observe, touch the book, and contemplate how to adapt the fairytale story to their personal experience.

The protagonist of the tale continues his journey, holding the shadow by the hand, while the person who contemplates his story activates mental images (Carston, 2010, 2018) that lead to understanding the true meaning of the stitched work: becoming an adult means being aware of oneself and accepting human weakness. The book can be interpreted as a work that the viewer can see now as one thing, now as another (Wittgenstein, 1953); thus, the pictures illustrated can be interpreted metaphorically. The black-colored stitched figure, which expands out of proportion until it takes on the appearance of a dragon, represents the dark side of man, while the celestial-colored figure defies the darkness in the guise of a hero–warrior.

The work requires a close distance to involve the viewer: “The thread indicates a relationship that unites and the fabric, the intersection of opposites (warp and weft), the creative dimension par excellence, conveys touch, the utmost closeness (touching creates intimacy)” (Cuccu, 2014: introduction, our translation).

The words in a book must be revitalized by those who read them; this revitalization similarly applies to works of art, which seek the gaze of the observer for interpretation and to gain new meanings over time. The multimodality of the stitched fairy tale (“After all, life is a continuous stitching,” as Maria Lai herself said) stimulates a path of identification in those who benefit from the vision and allows the emotional experience to be processed, no longer by the child but by the adult, as if it were a dream. Lai's stories are similar to variants of a single fairytale: that of art, which plays with archetypes (mystery, astonishment, fear, happiness, and magic) through the symbolic synthesis of materials, shapes, colors, and rhythms. These artworks entrust the reader with the role of the protagonist, who must elaborate on the meaning and gain awareness of his existence to understand them effectively (Cuccu, 2014).

### 4.2. Space category

As observed, Maria Lai's stitched narratives develop in a visual time that becomes a weaving space that can be touched, and they encourage an unprecedented imaginative learning experience in those who contemplate them. Sardinia is an island rich in open spaces in nature; the openness of the landscape inspires the sense of freedom and creativity of Sardinian artists.

**Figure 3:**
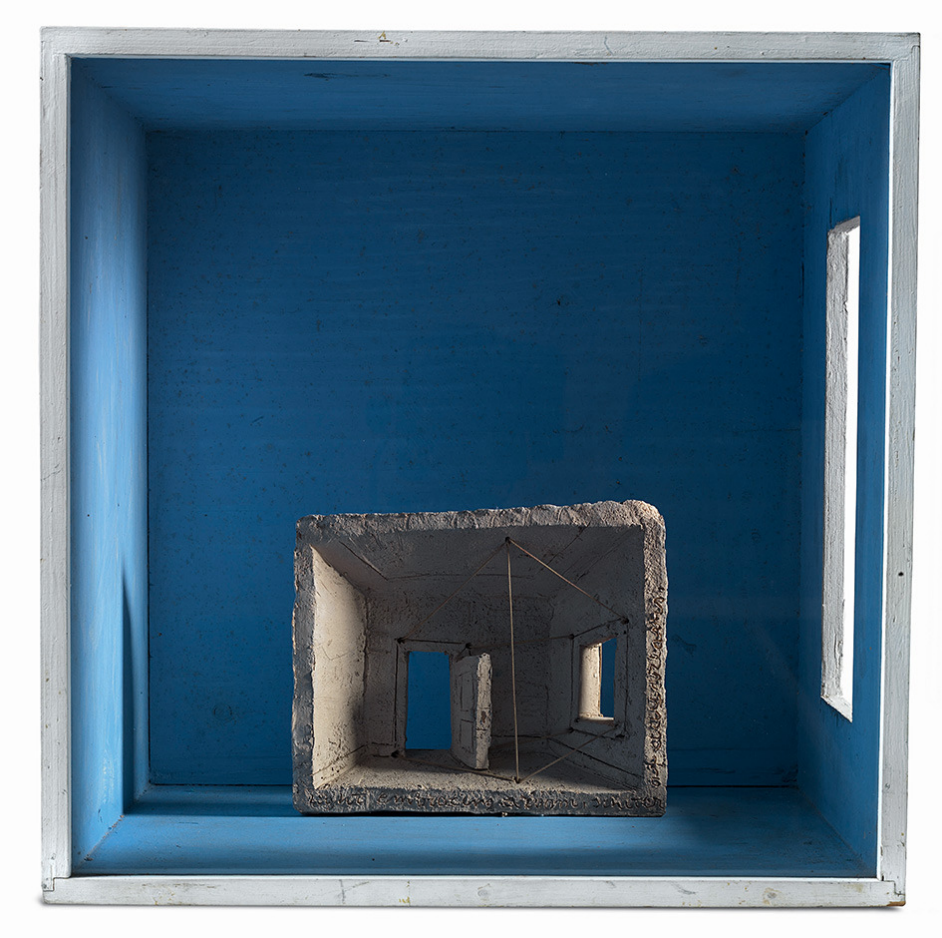
Costantino Nivola, *Light embracing a room, sinew of daydreams* (Orani, Museo Nivola)^19^ (reproduced with permission from Fondazione Nivola).

The multifaceted artist Costantino Nivola (1911–1988) experimented with the uses of the category “space” in various drawings, paintings, and sculptures during his years of research on the topic of rooms. Rooms are seen as geometric forms of a simple nature, open on the front side and equipped with small windows through which light enters and “modifies” the space. In the *architectural model*^18^ made in the 1980s, the title *Light embracing a room, sinew of daydreams (La luce abbracciando una stanza tende i sogni ad occhi aperti*) (Figure 3) determines the verbal–pictorial metaphor: the way spaces are constructed as the source domain and the symbolism generated as the target domain.

This work is presented as a kind of cube, open at the front, made of wood and glass painted in blue. Another geometric figure with a rough form is found inside, that is, a terracotta parallelepiped representing the interior of a room, whose perspective is investigated through the spatial directions suggested by the strings of ropes. The two rooms are structured according to the structure of the work within the work.

The entry of light through the window of the large room gives a diffuse luminosity that reverberates in the surrounding spatial configurations, modifying their nature. The window of the small room has received the light necessary for the new arrangement of its interior. The small open door, opening onto the blue background of the large wall, directs the gaze of the observer toward a wide vision and an imaginative construction comparable to a dreamlike horizon. These places constitute a symbolic space where one can find oneself and one's aspirations, or even recover memories of the past:

“Inside this room, I shall find/what I have/Always searched for, all variants of the/Square and/of the cube. The infinite linear variations/Of/The vertical and horizontal./In all the degrees of the angle I shall/Find/Myself as sole organic form. In this/Temple/Of Cartesian structures suspended in the/Chaotic context/Of infinite rifts”^20^.

The architecture of the spaces constructed in these rooms does not allow for an easy perceptual identification with the usual way of understanding and visualizing a room that is furnished and functional to our needs. The image has hidden properties because it evokes a dream world and triggers childhood memories in the observer. Thus, the observer activates the imagination to understand the meaning of the work and to discover those image properties that are not observed (Ervas, 2019). The taut cords connecting the lintels of the window and door to the ceiling and floor of the small room might suggest the idea of tending and turning in a direction; in a figurative sense, these cords trigger in the observer the mental image of “tending toward a goal, toward good or happiness”.

**Figure 4:**
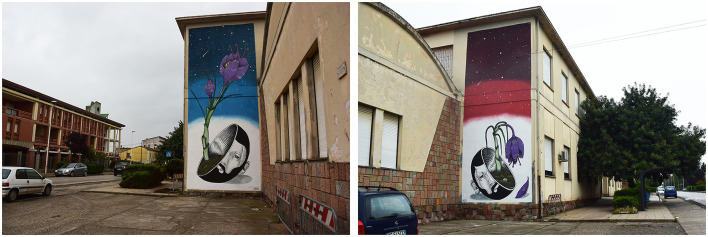
Andrea Casciu, *Crocus* (reproduced with permission from Andrea Casciu).

The category of “space” can also be interpreted as the source domain of an artistic metaphor. For instance, in the work *Crocus* by Andrea Casciu (Figure 4), the cycle of life and death is expressed through the saffron flower (the target domain) and the arrangement of the murals in the urban spaces of San Gavino Monreale. On the right side, facing the town center, the crocus sprouts inside the face of a man with a beard (the artist himself). On the left side, facing the road leading to Sardara, the flower withers (Fois, 2016). Thus, the mural depicting the birth of the saffron flower is located in the entrance area of San Gavino, while the one showing the withering flower is located in the exit area of the village.

The meditation on the nature of human beings and their transience is metaphorized in the representation of the body (Wollheim, 1984), that is, the half of the artist's skull that blossoms and fades similarly to a flowering plant. The observer could see saffron, the symbol of San Gavino peasant culture, as an aspect that contributes to the experience of vision (Wittgenstein, 1953) and knowledge of the artistic world. The two parts of masonry are conceived as a unitary work: as an extended metaphor in a narrative that needs the mural marking the entrance to the town and that marking the exit from the town to be completed.

Thus, the spaces conceived are part of the tradition of Sardinian muralism, understood as a form of public art that is “highly civic” (Naitza, 1996) and as a form of multimodal and intertextual expression (Piga, 2021) in the redevelopment framework of urban contexts following the most recent trends in Italian street art (Serra, 2007).

Furthermore, spaces that have been made functional again can contribute to the rediscovery of agricultural traditions in small villages. The town of Escolca, which is located on the basaltic plateau of the Giara di Serri in the Sarcidano (south-central Sardinia), has used urban art to promote its traditions of olive and durum wheat cultivation.^21^ The Fondazione di Sardegna, in collaboration with the Municipality of Escolca and the local associations, promoted the street art project *Codice colore 8015* (#codicecolore8015), in which five artists^22^ were called upon to intervene with works that reflect the identity of the village and the colors prevalent in step with the landscape context.

**Figure 5:**
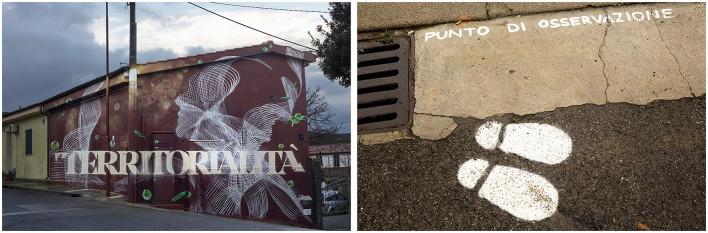
Manu Invisible, *Territorialità*^23^ (reproduced with permission from Manu Invisible).

In the work *Territoriality* (*Territorialità*) (Figure 5) created by one of the curators of the initiative, Manu Invisible, the artistic metaphor is presented in a multimodal format (a verbo-pictorial metaphor) (Forceville, 1996). The source domain is expressed in the representation of the visual space that encompasses the buildings and elements of the surrounding environment, whereas the target domain is made explicit by the concept of territoriality and verbally represented.

*Territoriality* reflects on the relationship between man and environment, focusing on the necessary rediscovery of balance between people and places, and is inspired by the reflections on human geography formulated by Claude Raffestin and centered on the concepts of society, space, and time (Klauser, 2012). The painted space emphasizes the elements of the cultivated land, expressed in the brown background, and the olive tree depicted in its symbols, olives, and leaves. The artwork is in a harmonious fusion with the individual, whose silhouette comprises remarkably thin threads as if superimposed in a criss-cross of layers to evoke the texture of the prized pasta from Nuoro, Filindeu. Thus, the artistic language incorporates important values (Gombrich, 1963), and those of rural tradition, such as the genuineness of flavors and the bond between the land and its inhabitants, are to be handed down to new generations. The action performed on a real territory closely involves the observer, who is invited to grasp the true essence of the meaning that the artist intends to convey. Near the mural, painted footprints are found on the ground to mark the point from which the spaces of the work can best be observed. Physical presence and an attentive gaze are important for the elaboration of the visual interpretative content; owing to their imagination, observers can overcome the limits set by the pictorial representation. The artist has also painted a lamppost and a road sign, becoming an integral part of the same territory that is the subject of the work.

### 4.3. Decontextualization of stereotypes category

The category of “space” can be expressed in a decontextualized way and with re-used materials that subvert the pre-constituted way of thinking of the observer: thus, a context such as the mines of Sulcis Iglesiente can be understood as a stereotypical space to be associated with Sardinia and as a real place to be represented beyond the boundaries set by figurative customs.

In the triptych *From Darkness to Light* (*Dall'oscurità alla luce*) by Paolo Piria,^24^ the re-used materials^25^ from the Monteponi mine are placed in a decontextualized canvas space. The access ladder to the shafts protrudes from the provided space and leads nowhere, the ice axe is dirty from use, and the helmet with the light bulb has lost its function (Guerrieri, 2021). In terms of an artistic metaphor, the objects and materials representing the mining world could be understood as the source domain, while the unframed canvas could be observed as the target domain. Therefore, these objects produce a particular viewing experience in the observer, who does not expect to see the symbolic elements of the mining collective imagination outside the confined landscape of the mines themselves. To provide a correct assessment of these elements, observers can bring their imaginative abilities into play and perform a critical process that leads to the discovery of those properties present in the image but ignored, such as the tactility of the materials. Even the reduced correspondence to the functions for which the objects are intended can invite the viewer to make explicit further components of the image's meaning, such as the dreamlike association between the color of the helmet and the gold symbol of every mineral seeker's dream. In the work, the three-dimensional component of the sign and the chromatic research suggest a unique fruition that amplifies the gaze and involves the external observer in the mining narrative.^26^

**Figure 6:**
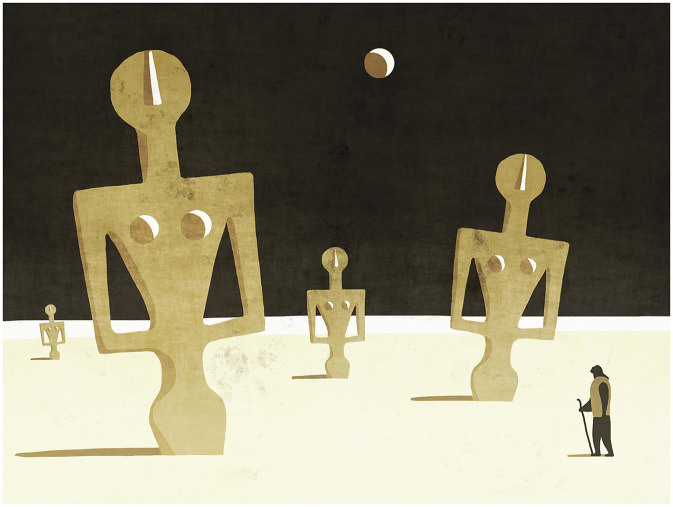
Toni Demuro, *Untitled* (reproduced with permission from Toni Demuro).

The category of decontextualization of stereotypes can also be observed within the boundaries of the painting, as in the illustration by Toni Demuro (Figure 6). In this artistic metaphor, the source domain could be represented by the Idols of Porto Ferro, the archaeological artifacts depicting the mother goddesses, while the target domain could be represented by the lunar setting. The two domains are unpredictably juxtaposed in an incongruous context; thus, the observer does not expect to find the artifacts in a context other than the museum. In the second stage of observation, the interpreter can make a visual-mental association between the arrangement of the idols as a flock and the presence of a shepherd guiding them; thus, the sheep can be understood as an aesthetically relevant property in the image (Nanay, 2016; p. 67). In this prototypical sense, sheep as animals stereotypically identified with Sardinia activate another mental image connected to the idea of holiness. Simultaneously, the layering of hidden and implicit meanings in the work can generate a further interpretive process linked to the construction of (decontextualized) stereotypes. The mental image of the sheep can be oriented in a stereotypical sense and lead the mind to an erroneous perception of a specific community: the Sardinians belonging to the category of shepherds are usually (and wrongly) interpreted from an outside perspective as a condition of subservience and backwardness. Even the depiction of idols extracted from their sphere of reference can activate stereotypical interpretations linked to the socio-historical imaginary. Idolatry toward these objects could suggest a deep identification of the Sardinian people with their archaeological heritage, almost as if this was the only one worthy of being divulged outside the territory of the island. The identification is also with its (false) myths, that is, the predominant presence of a sort of “female element” in all the historical and social expressions of Sardinia (Melis, 2015). The perception of the difficulty in imagining a different way of representing oneself and making oneself known outside one's own borders emerges and could also lead to closure within oneself and to that “lunar” feeling of loneliness with a “dechirican” air that permeates the work of Demuro.

### 4.4. Fusion of forms category

In the fourth macro-category, the “fusion of forms” is achieved through an expressive technique or the use of color.

In the artistic metaphor by Salvatore Aste,^27^ the source and target domains are merged to reveal the metaphorization of physical forms. The artist paints the curves on the outside of the figure with shaded brushstrokes in black, purple, and burgundy. In addition, this painted surface includes the pearl-colored inner section, which the interpreter assimilates in shape and color to a bivalve (to be understood as the source domain).

In the physicality (Wollheim, 1984) of the latter, the observer can identify a humanized figure with delicate feminine features, an elongated face, and narrow eyes (similar to the long-necked women portrayed by Amedeo Modigliani), to be understood as the target domain of the metaphor.

Associating the female gender with the mussel, commonly called “cozza” in Italian, is not meant to be derogatory, as clichés often invite one to do, but rather to identify in the canvas a sophisticated symbiosis between the individual and nature. Indeed, both come from the same matrix.

Identifying new elements in the image is possible by analyzing the title of the work. Some studies have shown that titles are not mere identification tags but rather have the function of orienting the interpretation of the observer (Fisher, 1984; Levinson, 1985) or guiding the gaze of the viewer in a different way, especially in the case when the titles of certain works are subject to variation (Franklin et al., 1993)^28^. Thus, the canvas painted by an artist from the Island of San Pietro (Carloforte) and titled *Woman in the Mirror* directs the gaze: the pearly-colored figure with eyes, mouth, nose, and flowing hair is clearly a woman looking into the mirror and perhaps turning into something else, as the black background could be interpreted as a mysterious vortex in which the figure appears to be enveloped.

## 5. Conclusion

The multimodal power of metaphor in art has been investigated in this study *through* four categories: (1) time, (2) space, (3) decontextualization of stereotypes, and (4) fusion of forms. Of course, this is a first attempt to categorize artistic metaphors, which might be limited, and further categories may be added in future research.

The evocative power of Sardinian art expressed through sculpture, weaving, painting, mural and poly-material art, architecture, and illustration proves that an island is a fertile place of creativity. Therefore, the visual component stimulates knowledge of the “submerged” wealth of symbols, archetypes, and myths that characterize insularity.

By contrast, the term “image” can encompass a range of meanings: it extends to the domain of material images on a specific media support or to that of immaterial images, on whose borders live dreams, ideas, and metaphors; image as a product of a mind that thinks, remembers, or dreams; as a synonym of the artistic image; and as an element that triggers connections between subjects and dialogical relations (Crescimanno, 2019; pp. 24–25).

The multimodality expressed by the categorization of artistic metaphors experimented on a small corpus of Sardinian works can distance observers from erroneous or stereotypical perceptions regarding the island context and direct them to surprising learning perspectives.

## Data availability statement

The original contributions presented in the study are included in the article/supplementary material, further inquiries can be directed to the corresponding author.

## Author contributions

AG wrote the article. FE and EG supervised the work. All authors provided feedback on the draft and approved the final version of the manuscript.

## Footnotes

^1^Image reference: <https://it.wikipedia.org/wiki/Illusione_anatra-coniglio#/media/File:Kaninchen_und_Ente.png> (09/01/2022).

^2^Reference to the image: https://www.tate.org.uk/art/artworks/blake-the-river-of-life-n05887 (02/16/2023).

^3^Critical references to the study are at the following link: <https://www.moma.org/audio/playlist/243/3126> (09/01/2022).

^4^Critical references to the study: <https://www.moma.org/learn/moma_learning/marina-abramovic-marina-abramovic-the-artist-is-present-2010/> (09/01/2022).

^5^An image of the artwork can be found at the following address: <https://it.cathopedia.org/wiki/File:Giotto_-_Legend_of_St_Francis_-_-03-_-_Dream_of_the_Palace.jpg> (09/01/2022).

^6^Reference to the image: < https://www.moma.org/collection/works/79018> (02/12/2023).

^7^Reference to murals: https://www.haring.com/!/genre/public_projects (02/12/2023).

^8^Reference to *La condition humaine* (1933), https://www.nga.gov/collection/art-object-page.70170.html#provenance and La condition humaine (1935), https://www.wikiart.org/en/rene-magritte/the-human-condition-1935 (02/12/2023).

^9^Image references: <https://artsandculture.google.com/asset/the-scream-edvard-munch/eQFdRTFKDtVQ1A> (09/01/2022).

^10^Image references: https://it.wikipedia.org/wiki/Guernica_(Picasso)#/media/File:Mural_del_Gernika.jpg (09/01/2022).

^11^Reference to the study *Sacco Rosso*: <https://www.tate.org.uk/art/artworks/burri-sacking-and-red-t00787> (09/01/2022).

^12^Image reference: <https://artsandculture.google.com/asset/bgEuwDxel93-Pg> (09/01/2022).

^13^Image reference: <https://en.wikipedia.org/wiki/The_Broken_Column> (09/01/2022).

^14^With reference to what Sciola said during an interview for *Born of Stone* (2016). *Born of Stone* is a short documentary about Pinuccio Sciola directed, edited and produced by Emilio Bellu. The movie is available on the ITsArt platform: https://www.itsart.tv/it/content/born-of-stone-2016/c42ca446-0224-4aaa-87b2-37ccf61dfc59?hl=en-IT, (03/12/2023).

^15^Image and acoustic references in the short movie *Sciola, oltre la Pietra* (2009) directed, edited and produced by Franco Fais: <https://www.youtube.com/watch?v=lkq33RNZpL4> (03/12/2023).

^16^Image reference: < https://marianneboeskygallery.com/artworks/20341-maria-lai-libro-delle-formiche-1991-1992/> (02/12/2023).

^17^Image reference: < https://catalogo.beniculturali.it/detail/SARDEGNA/HistoricOrArtisticProperty/2000220889> (09/01/2022).

^18^Critical reference in Nivola (2010). ‘L'investigazione dello spazio. Un itinerario', in *NIVOLA. L'investigazione dello spazio*, ed. C. Pirovano (Nuoro, TRIBU piazza Santa Maria della Neve, 22 dicembre 2009 - 21 giugno 2010), 160-163.

^19^Notation: painted wood and glass, 40.8 × 40.8 × 40.8 cm; inside: semi-refractory clay with cotton cord, 17 × 20.4 × 5 cm.

^20^Homage to Marcel Proust, composition written by Costantino Nivola. Reference to the website dedicated to the artist https://museonivola.it/en/collection/ (09/01/2022).

^21^See: Associazione culturale Asteras (2020). *Muri di Sardegna: luoghi e opere della Street art*. Palermo: Flaccovio editore, 131.

^22^In addition to Manu Invisible, Davide Medda, Mavz, Lorenzo Murgia, and Davide Careddu participated in the initiative.

^23^Notation: (silossanica) paint and spray on wall, electrical box and pole 45 mq.

^24^Image reference in https://issuu.com/planumnet/docs/esplorazioni_narrative_artistiche_e_videoludiche./s/15183014 (02/12/2023).

^25^Earth, stones, pink mud, ladder to access the wells, ice axe, helmet with bulb.

^26^Audiovisual reference: https://www.youtube.com/watch?v=bg6UHeySJ7U (09/01/2022).

^27^Image reference: https://www.isoladisanpietro.org/cnm/pennellate_tabarchine/salvatore_aste/quadri/07.htm (09/01/2022).

^28^In this research, it was shown that the images presented to observers (those of the paintings *The Terrace at Sainte-Adresse* by Claude Monet and *Agony* by Arshile Gorky) are described differently when they have different titles (*Imminent Storm* and *Carnival*, respectively).
